# A promising target for breast cancer: B7-H3

**DOI:** 10.1186/s12885-024-11933-3

**Published:** 2024-02-07

**Authors:** Ying Jiang, Jiayu Liu, Lingyan Chen, Zhiwen Qian, Yan Zhang

**Affiliations:** 1 Department of Oncology, Wuxi Maternal and Child Health Care Hospital, Women’s Hospital of Jiangnan University, Jiangnan University, Wuxi, 214002 China; 2 Wuxi Maternal and Child Health Hospital, Nanjing Medical University, Wuxi, 214000 China

**Keywords:** Breast cancer, B7-H3, Immune checkpoint, Cancer immunotherapy, Tumor microenvironment, Targeted immunotherapy

## Abstract

Breast cancer (BC) is the second-leading factor of mortality for women globally and is brought on by a variety of genetic and environmental causes. The conventional treatments for this disease have limitations, making it difficult to improve the lifespan of breast cancer patients. As a result, extensive research has been conducted over the past decade to find innovative solutions to these challenges. Targeting of the antitumor immune response through the immunomodulatory checkpoint protein B7 family has revolutionized cancer treatment and led to intermittent patient responses. B7-H3 has recently received attention because of its significant demodulation and its immunomodulatory effects in many cancers. Uncontrolled B7-H3 expression and a bad outlook are strongly associated, according to a substantial body of cancer research. Numerous studies have shown that BC has significant B7-H3 expression, and B7-H3 induces an immune evasion phenotype, consequently enhancing the survival, proliferation, metastasis, and drug resistance of BC cells. Thus, an innovative target for immunotherapy against BC may be the B7-H3 checkpoint.

In this review, we discuss the structure and regulation of B7-H3 and its double costimulatory/coinhibitory function within the framework of cancer and normal physiology. Then we expound the malignant behavior of B7-H3 in BC and its role in the tumor microenvironment (TME) and finally focus on targeted drugs against B7-H3 that have opened new therapeutic opportunities in BC.

## Introduction

BC is the leading cause of disability and death among women globally [1]. The World Health Organization reports that approximately 2.26 million women are given a BC diagnosis every year [1]. Mosaic populations of tumor cells, immune cells, and stromal cells that have different genetic, epigenetic, and phenotypic traits make up breast malignancies. Four molecular subtypes of BC were categorized by gene expression sequence analysis; these include Luminal A, if estrogen receptor alpha-positive (ER) + and/or progesterone-receptor (PR) + , human epidermal growth factor receptor 2 (HER2) − , Ki67 < 14%), Luminal B (if ER + and/or PR + , HER2 overexpressed or Ki67 ≥ 14%), triple-negative breast cancer (TNBC) (if ER − , PR − , HER2 −), and HER2-enriched (if ER − , PR − and HER2 +) [2]. The specific receptors that cancer cells express (or do not express) act as biomarkers for therapy. Anti-estrogens and aromatase inhibitors, both of which disrupt ER activity, are effective against ER-α positive cancers [3]. Therapeutic agents directed at HER2, such as trastuzumab—an anti-HER2 antibody—demonstrate anticancer efficacy specifically in HER2-positive malignancies [4]. Hormone-responsive BC has been successfully treated with endocrine treatment. Regretfully, disease recurrence and relapse are caused by the emergence of drug resistance [5], TNBC has the poorest prognosis because of the high intra-tumor heterogeneity and absence of specific receptors [6]. Therefore, the outlook for women with BC remains grim. The immune and stromal cell subsets that compose the breast tumor ecosystem are extremely complicated, and their makeup, spatial arrangement, and functional orientation all significantly impact how the illness develops and how patients fare. Consequently, it is crucial to establish effective BC treatment techniques and identify new therapeutic targets. Cancer treatment has undergone a paradigm shift as a result of recent developments in immune checkpoint inhibitor (ICI) medicines [7].

Particular focus has been placed on the B7 family proteins due to its potential use as an ICI to cure cancer. Members of the B7 family closely regulate immunological responses [8] and tumor progression [9]. The 10 members of the B7 family that are now recognized include B7-1/CD80, B7-2/CD86, B7-H1/PD-L1, B7-DC/PD-L2, B7-H2/CD275, B7-H3/CD276, B7-H4/VTCN1, B7-H5/Vista, B7-H6/NCR3LG1, and B7-H7/HHLA2 [10]. It has been demonstrated that B7-H1/PD-L1 and B7-DC/PD-L2 interact with PD-1 (programmed death 1) and stimulate the growth of T cells via secreting IL-10 and interferon-γ [11]. In contrast, the T-cell response is inhibited and immune evasion is facilitated when PD-L1 is expressed on cancer-associated cells [12]. PD-1/PD-L1 pathway proteins have been targeted by antibodies to treat a variety of malignancies [13]. However, certain tumors that exhibit high PD-L1 proteins were found to respond to PD-L1 treatment with a low objective response rate (ORR), likely because the TME significantly affects how well the immune system responds to these inhibitors [14–16]. Just 40% of patients have clinically reacted to PD-1/PD-L1 blocking [17]. Thus, it is crucial for therapeutic purposes to find new biomarkers in patients who respond to ICIs.

Among B7 family members, B7-H3 has recently received attention because it is significantly expressed in several malignancies and predict a dismal prognosis [18–22]. The expression of B7-H3 on the surfaces of tumor cells stimulates the growth of tumors by allowing these cells to evade immunosurveillance [23]; Compared to normal tissues, tumor tissues have an excessive expression of B7-H3 [24, 25]. The American Joint Committee on Cancer evaluated B7-H3 expression in stage I to III primary breast cancer and normal breast specimens, results showed that 39% of initial breast cancers had B7-H3 mRNA expression, whereas normal breast tissues did not [26]. Moreover, B7-H3 was substantially linked with tumor formation and lymph node metastasis in primary breast cancers [26]. Elevated expression of B7-H3 was tied to a worse prognosis in a five-year examination of BC patients’ survival rates [27] and bad clinicopathological BC parameters [28]. According to another research, individuals with BC who have high levels of B7-H3 expression in their circulating epithelial tumor cells are more likely to develop metastases [29]. Hence, we propose that the B7-H3 immune checkpoint may be a promising target in BC immunotherapy.

## B7-H3’s structure and physiological implications

B7-H3 is a dual-acting immunological checkpoint protein that is expressed on cancer cells and antigen-presenting cells (APCs) including dendritic cells and macrophages. It is effective in both soluble and membrane-associated forms [30]. The soluble form can be produced by selective splicing [31] or, more commonly, by cleavage of B7-H3 present on the surfaces of monocytes, DCs, and T cells by membrane metalloproteinases [32]. The membrane-associated form has an extracellular Ig-like structural domain, a transmembrane part, and a shorter intracellular region [33]. The number of extracellular Ig-like domains that each of the two membrane-bound B7-H3 isoforms, 2IgB7-H3, and 4IgB7-H3, contains serves to distinguish them from one another; the former contains a single IgV (variable) domain and a single IgC (constant) domain, due to exon duplication, the latter has tandemly duplicated IgV and IgC domains [34]. B7-H3 has both stimulatory and inhibitory properties to increase or decrease the activity of T cells, possibly due to its interaction with various receptors that have different functions in specific contexts. However, the B7-H3 receptor’s identification is up for debate. Certain putative receptors, including phospholipase A2 receptor 1, interleukin-20 receptor subunit α, and the trigger receptor expressed on myeloid cells-like transcript 2, have not been conclusively verified [35]. The unknown nature of the B7-H3 receptor has been a major obstacle to understanding the biology of B7-H3. Although considerable efforts have been made to solve this problem, the available data on the B7-H3 receptor remain contradictory and limited. In addition to its immunological activity, B7-H3 is known to be essential for maintaining the balance between osteoclast and osteoblast growth [36, 37]. Moreover, B7-H3 knockout mice often have alterations in oxidative phosphorylation and poor fat storage, leading to spontaneous obesity [38].

## Regulation of B7-H3

Nonregulatory expression of B7-H3 in a spectrum of malignant cancers has been observed and correlates with a poor prognosis [39–42]. Protein-level expression profile of B7-H3 indicates that posttranscriptional and posttranslational regulations are essential for its expression, and the effect of modulating B7-H3 expression on BC is progressively being investigated. Through interacting with the 3'-untranslated region of B7-H3, miR-29c tightly controlled B7-H3 to lower its expression in BC tumors [43]. Alternative splicing is a crucial process for regulating gene expression and producing proteome diversity [44]. Both 4IgB7-H3 and sB7-H3 are produced by alternative splicing. Scientists have investigated the employing of sB7-H3 in the diagnosis of BC by evaluating serum sB7-H3 levels by ELISA, using healthy subjects and benign breast disease (BBD) patients as controls. Individuals with BC reported significantly higher sB7-H3 levels than controls; thus, sB7-H3 may be a potential biomarker that can be applied to distinguish individuals with BC from healthy individuals and those with BBD [45]. Glycosylation, a posttranslational alteration that regulates the solubility, structure, and function of proteins, is crucial for biological function [46]. In TNBC patients, the fucosyltransferase FUT8 stabilizes and encourages high B7-H3 production via regulating B7-H3 core fucosylation, the B7-H3 protein’s glycosylation may serve as a poor predictive indicator of survival [47].

## B7-H3’s potential contribution to breast cancer

**Fig. 1:**
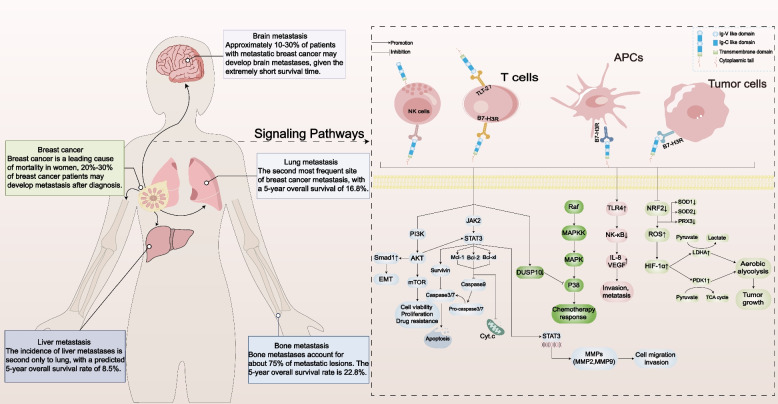
An overview of the molecular pathways behind B7-H3’s tumor-causing activities. When B7-H3 is expressed on the cell membrane, it initiates multiple signaling cascades, activating downstream molecules and facilitating the malignant tendencies of cancer cells

It has been demonstrated that B7-H3 is involved in several tumor-related activities. The relationship between B7-H3 and tumorigenesis, as well as the signaling pathways through which it operates, will be detailed below (Fig. 1).

### B7-H3 and breast cancer proliferation

BC is believed to be a stem cell disease because it contains cancer cells that resemble stem cells and have tumor-causing capacity [48]. These cells are in charge of the formation and metastasis of malignancies. Researchers have recently found that BC stem cells can be created from nonstem cells, cancer stem cells (CSCs) and cancer cells undergo a biological transition that keeps the cell population in balance [49]. CSCs are a tiny subpopulation of cancer cells that control resistance, metastasis, recurrence, and invasiveness in tumors. Cancer can develop when a self-replicating stem or progenitor cell undergoes a malignant change [50, 51]. Al-Hajj et al. were the first to note the existence of CSCs in BC; they successfully induced xenograft tumors to grow in immune-deficient mice [52]. Small quantities of breast cancer stem cells (BCSCs) can generate tumors, and they are resistant to chemotherapy and radiation [53, 54]. Similar to stem cells, BCSCs regulate self-renewal and differentiation in a sensitive way to preserve tumor homeostasis [55]. On the one side, they transform into various cancer cells that combine to form the tumor mass. On the other side, when cancer spreads, they continue to replenish the stem cell pool [56]. Several attempts have been taken to eliminate BCSCs because of their significant contribution to cancer growth.

One investigation found that B7-H3 expands the number of BCSCs by activating MEK through MVP [57]. MEK activation is required to maintain the viability of cancer stem cells [58]. Additional evidence for this view is antibodies against B7-H3 eliminated cancer stem cells and prevented tumor development in a way that was CD8 + T-cell reliant [23]. Our knowledge of BCSC biology and normal stem cell biology is expanding, and B7-H3 might be employed as a target to prevent the growth of tumors and BCSCs.

### B7-H3 and breast cancer metastasis

BC patients who do not develop metastases have a 5-year overall survival rate of more than 80% [59], and distant metastasis may result in a sharp decline in 5-year overall survival to just around 25% [60]. For HER2 + or ER + subtypes of metastatic BC, the median overall survival is around 5 years; for triple-negative subtypes, it is 1 year. Presently, almost all patients with metastatic BC still have no chance of recovery [61]. Breast cancer frequently spreads to several organs, including the bone, lung, liver, and brain; metastatic heterogeneity refers to this phenomenon, which causes individuals to respond to therapy differently and has diverse prognoses. Among all metastatic lesions, bone metastases comprise around 75% [62], and patients with bone metastases had a 5-year overall survival rate of 22.8% [63]. The second most typical location for the spread of BC is the lung, and patients who experience such metastases have a 16.8% five-year overall survival rate [64]. While liver metastases are less common than lung metastases, their expected 5-year overall survival rate of 8.5% is lower than that of individuals with lung cancer, local–regional cancer, or bone cancer [65]. Brain metastases occur in 10–30% of individuals with metastatic BC; given the extremely short survival time, these metastases represent a significant quality and length of life limitation for many patients [66, 67]. There are still many unknowns about the multiphase metastatic process, despite decades of study on metastasis giving us tremendous understanding. Therefore, a systematic and in-depth survey into the mechanisms of BC metastasis and the exploration of effective metastasis-targeting drugs is necessary.

Strong evidence linking B7-H3 expression dysregulation to cancer metastasis has been found [40, 68, 69]. B7-H3 was found to mediate tumor metastasis through several signaling pathways. JAK/STAT signaling and the downstream effectors of this pathway: SLUG, MMP-2, and MMP-9, have been associated with B7-H3-mediated metastasis [70]. By downregulating metastasis-related proteins such as MMP-2, STAT3, and IL-8, B7-H3 promotes the motility and invasiveness of tumor cells [70, 71] and through the JAK2/STAT3/MMP-9 signaling pathway [72]. In addition, it was discovered in one study that B7-H3 activates the PI3K/AKT pathway to increase the expression of SIRT1 and that it further promotes E-cadherin expression and epithelial-mesenchymal transition (EMT) [73]. A crucial stage in the spread of cancer is the EMT. In another study, by triggering the PI3K/AKT and p38/ERK MAPK signaling pathways, B7-H3 has been reported to support the EMT process [74]. Finally, B7-H3 increases the production of IL-8 and VEGF and activates the TLR4/NF-κB signaling pathway to promote metastasis [75]. Although the mechanisms they reveal are diverse, tumor invasiveness and metastasis have been demonstrated to be impacted by B7-H3. Investigation of the mechanisms through which B7-H3-promoted metastasis occurs makes it possible to target the pathways involved and thereby attenuate BC metastasis via interfering with signals mediated by B7-H3.

### B7-H3 and breast cancer metabolism

Regardless of the abundant availability of oxygen, tumor cells preferentially use glycolysis to produce energy and therefore exhibit a higher rate of glycolysis than other cells. Aerobic glycolysis was first described by the German biochemist Otto Warburg and is known as the Warburg effect [76]. Aerobic glycolysis is a scientifically recognized feature of cancer cell metabolism, and the Warburg effect has been shown to occur in BC [77]. Several studies have shown that exploring ways to suppress the Warburg effect could be instrumental in the fight against BC, including refractory phenotypes TNBC [78–82]. There is evidence that B7-H3 promotes glucose absorption and tumor development in BC [83]. B7-H3 increases reactive oxygen species (ROS) to support HIF-1a stability by suppressing the activities of the transcription factor NRF2 and NRF2 antioxidant targets SOD1, SOD2, and PRX3, thus boosting expression of the glycolytic enzymes LDHA and PDK1; this inhibits pyruvate transit through the TCA cycle while promoting the transformation of pyruvate into lactate [83]. Decreased B7-H3 expression in TNBC cells showed a reduced rate of glycolysis and better sensitivity to AKT/mTOR inhibitors [84]. Blockade of B7-H3 probably affects glucose metabolism through cellular ROS signaling and shifts the cell’s metabolic process from glycolysis to oxidative phosphorylation [85]. These results provide strong evidence for the role of B7-H3 in carcinogenesis and the deregulation of cancer cell metabolism.

### B7-H3 and breast cancer drug resistance

Several BC patients demonstrate inherent drug resistance, while others are initially drug-sensitive but develop resistance to anticancer treatments and commonly display multidrug resistance, which may cause recurrence and/or metastasis even though the prognosis for BC patients has significantly improved [86–88]. Nowadays, medication resistance is a significant factor in poor prognosis, lowering BC patients’ survival rates [89]. Hence, enhancing BC’s chemosensitivity would be a crucial stage toward better treatment of this condition.

Some preliminary evidence indicates that B7-H3 influences DNA repair processes or cancer cell stemness and thereby affects chemoresistance [57, 90]. It has been shown to encourage resistance to traditional cancer treatments in certain disease types [91–95]. Many currently undefined mechanisms may be involved, and a deeper understanding of how B7-H3 increases medication resistance might result in the creation of more potent treatments. Liu et al. found that B7-H3 is crucial for controlling the Jak2/Stat3 signaling pathway; this pathway at least partially induces paclitaxel resistance in breast cancer cells [96]. When B7-H3 is silenced, Jak2 and Stat3 are less phosphorylated, which reduces the production of anti-apoptotic proteins Mcl-1 and survivin [96]. B7-H3 is also involved in the MAPK Raf/MEK/ERK pathway [40]. The MAPK pathways drive various cellular processes; four primary pathways are involved, each of which is defined by its MAPK effectors: ERK1/2, ERK5, JNKs, and p38 MAPK [97]. Chemotherapy resistance may arise due to the phosphorylation of numerous transcription factors activated by the p38 MAPK pathway [98]. The MAP kinase phosphatase DUSP10 is recognized for its role in negatively regulating and dephosphorylating p38 MAPK [99]. According to one research, the p38 MAPK pathway serves as a significant mediator of B7-H3-induced drug resistance, and they also discovered a novel B7-H3-associated regulation of p38 MAPK activation. This regulation appears to be partially facilitated by the downregulation of DUSP10 [100]. The research results substantiate the existence of a B7-H3-DUSP10-p38 axis. In this regard, it has been shown that inhibiting p38 MAPK makes BC cells more sensitive to taxanes [101]. In conducting drug screening with human TNBC cell lines, researchers discovered that under circumstances of reduced B7-H3 expression, the cellular response to API-2 (triciribidine) and everolimus (RAD-001), two inhibitors that target proteins in the AKT/mTOR pathway, was boosted [84]. These results suggest that B7-H3 lessens the susceptibility of tumor cells to various chemotherapeutic medications; therefore, it is a valuable target for boosting the effectiveness of conventional cancer therapy.

## B7-H3 in the tumor microenvironment

**Fig. 2:**
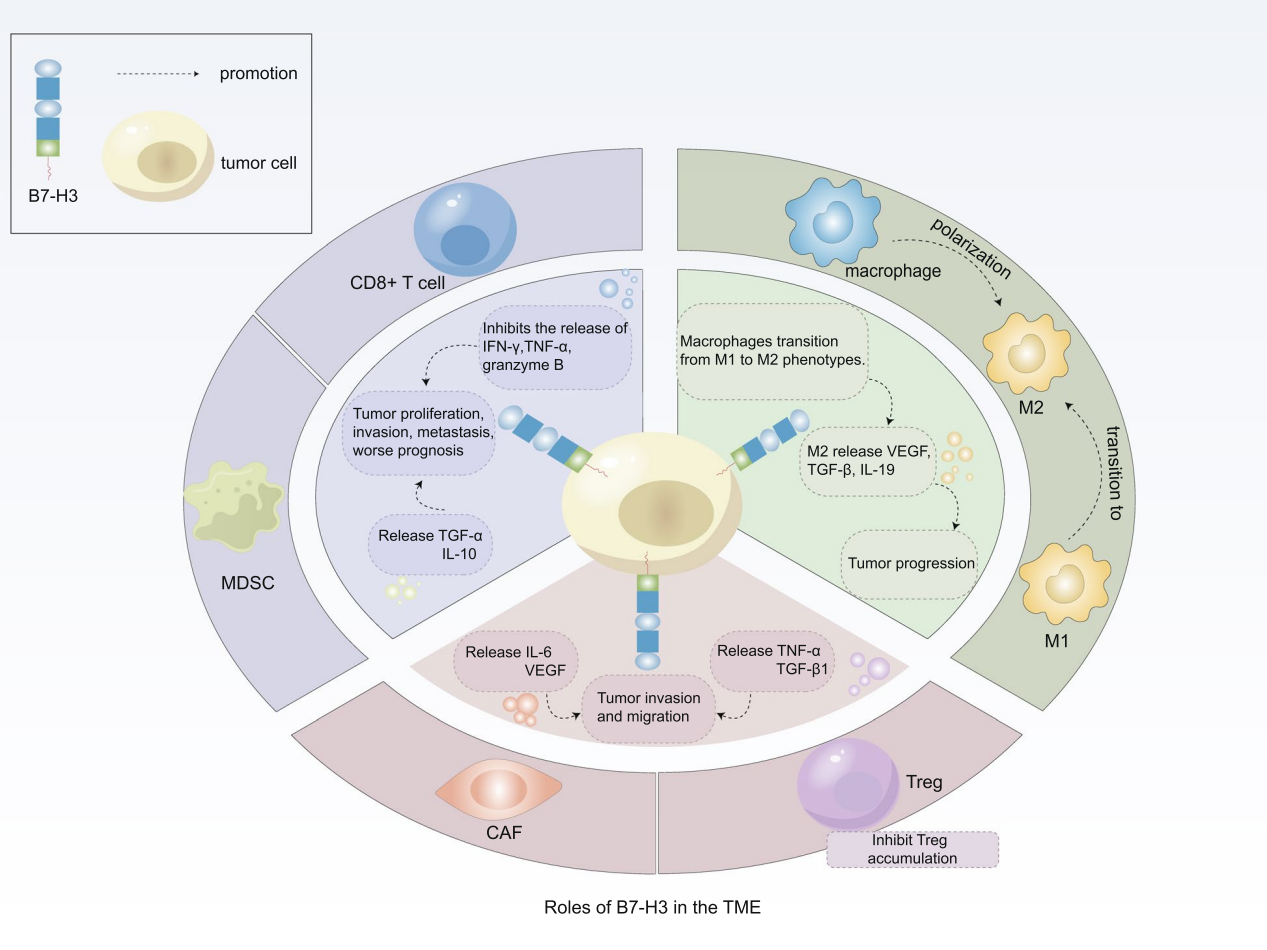
B7-H3’s function in the TME in conjunction with immune cells. B7-H3 modulates cytokine secretion in various types of cells within the TME, including T cells, macrophages, MDSCs, Treg, and CAFs. This contributes to the remodeling of the TME

Cancerous development occurs in a complex tissue environment that supports it. The TME contains stromal cell types that are genetically stable, as opposed to tumor cells. Many cell types, including immune cells, fibroblasts, and endothelial cells, comprise the tumor microenvironment [102]. The TME establishes a tumor-promoting “macroenvironment” that severely constraints cancer immunotherapy’s effectiveness [103]; therefore, specific disruption of the protumorigenic TME is an appealing therapeutic target, lowering the likelihood of tumor recurrence and resistance. To achieve this goal, a thorough comprehension of B7-H3 signaling becomes indispensable. Such understanding holds the key to the development of successful BC therapies by unraveling the intricate interactions among different components within the tumor (Fig. 2).

### MDSCs

Evasion and inhibition of the host immune system is a crucial stage in malignant tumorigenesis [104]. One of patients’ most common immune evasion mechanisms is suppression by Myeloid-derived suppressor cells (MDSCs). MDSCs are immunosuppressive, immature myeloid cells according to their functional definition [105]. Increased numbers of MDSCs in peripheral blood are observed in cancer [106]. MDSCs recruit T regulatory cells to boost immunosuppression further, while MDSCs directly repress natural killer (NK) and T cells [107]. During cancer development, MDSCs inhibit innate and adaptive immunity, lowering immune surveillance and preventing the immune system from destroying newly altered cells [108]. Moreover, they promote angiogenic activity, which aids in tumor invasion and spread [109]. Numerous cytokines are released by BC cells, including granulocyte–macrophage colony-stimulating factor, one of the main soluble BC-derived molecules that regulate the development of MDSCs from monocyte/granulocyte progenitors [110, 111]. The prevalence of circulating MDSCs in the peripheral blood of BC patients is increased at all phases of the illness. It positively correlates with the clinical stage and the burden of metastatic tumors [112]. MDSCs may inhibit the antitumor immune response by altering the expression of indoleamine-pyrrole2,3-dioxygenase (IDO), an enzyme in BC patients whose expression is correlated with lymph node metastasis [113]. In patients with TNBC, MDSCs were found to be critical negative modulators of antitumor immunity [114]. Targeting MDSCs may be a viable tactic for enhancing the efficacy of immunotherapy treatments since they pose a significant barrier to many cancer immunotherapies.

Zhang et al. discovered a unique subpopulation of B7-H3 + MDSCs that supports the development of tumors. To further characterize B7-H3 + MDSCs, the investigators examined the profile of cytokines produced by B7-H3 + MDSCs; The findings revealed that B7-H3 + MDSCs emit considerably more IL-10 and TNF- α than do other cells. Furthermore, B7-H3 + MDSCs were observed to cause amplification of Tregs, another possible mechanism through which tumor proliferation occurs [115]. Several studies of pulmonary fibrosis have shown that sB7-H3 causes MDSCs accumulation in the bone marrow and is associated with elevated inflammatory cytokine expression [116, 117]. The quantity of CD11b + Gr1 + MDSCs in murine lymphomas and subcutaneous melanoma tumors was significantly decreased by silencing of B7-H3 [118]. We hypothesize that MDSCs are a collection of immune cells that suppress the body’s defenses; in cancer, B7-H3 strengthens these cells’ inhibitory capabilities.

### TAMs

The most common immune cells inside the TME are tumor-associated macrophages (TAMs). Although macrophages are classically considered the critical effector cells of the immune defense, many studies have shown that TAMs assist the development of tumors in various ways [119]. A TAM signature for BC has been found that is considerably enriched in aggressive BC subtypes and is connected to a reduced disease-dependent survival rate [120]. In addition, TAMs pre-treated with IL-6 increased the growth and metastasis of TNBC cells and decreased their susceptibility to the chemotherapy drug cisplatin [121]. By paracrine signaling loops involving CSF-1 from the tumor and EGF from macrophages, TAMs may encourage tumor cell invasion [122–124]. Thus, regenerating TME-resident macrophages may have advantageous anticancer properties.

The different functions performed by macrophages in normal tissue homeostasis and cancer may be partly explained by their phenotype. Since they have functional plasticity, macrophages may alter their polarity to adapt to physiological circumstances. At the furthest points of their phenotypic spectrum [125], Macrophages polarize from the M1 state to the M2 state. Traditionally activated M1 macrophages produce type I proinflammatory cytokines, which have anticancer effects and are engaged in antigen expression. In contrast, Type II cytokines are produced by “alternatively activated” M2 macrophages, which contribute to the anti-inflammatory response and have a proliferative function in tumors. B7-H3 triggers the transition of TAMs from the M1 phenotype to the M2 phenotype [126]. B7-H3 was found to strongly regulate CCL-2 production through the STAT3 pathway and the CCR inhibitors partially abolish the impact of B7-H3 deletion on M2 macrophages, suggesting that the B7-H3-CCL2-CCR2 axis regulates TAM function [127]. It was shown that TAMs from patients with triple-negative BC had significant levels of B7-H3 expression. These B7-H3-highly expressing TAMs contribute significantly to metastasis and immunosuppression through inherent extracellular matrix (ECM) remodeling and tumor angiogenesis, changes that ultimately reduce T-cell invasion of the TME [128]. Investigating the signaling pathway through which these effects occur showed that upregulation of B7-H3 expression by lncRNA NEAT1 encourages M2 macrophage polarization through the JAK2-STAT3 pathway [129]. Using B7-H3 to change the behavior of TAMs provides a possible target for BC therapy.

### TILs

Type 1 T cells are related to a positive outlook in individuals with BC. By releasing cytokines and stimulating APCs, CD4 + T-helper 1 (Th1) cells support antigen presentation [130]. Forkhead Box P3 (FOXP3) CD4 + regulatory T cells are a subset of type 2 CD4 + T-helper cells (Th2) that suppress CTL activity, boost B-lymphocyte proliferation and may instigate an anti-inflammatory, immune response that encourages tumor progression [131]. CD8 + cytotoxic T cells (CTL) are vital for the elimination of tumors [130]. Immune cell infiltration of tumors has been proven to enhance prognosis in individuals with cancers [132–134]. Especially in BC, data suggests that the presence of tumor-infiltrating lymphocytes (TILs) prior to therapy predicts treatment response and is linked to a better prognosis [135, 136]. Different BC subtypes have diverse TIL ratios. For instance, the TIL percentage is higher in HER2 + and TNBC patients than it is in individuals with hormone receptor positivity [137], patients with high TIL ratios have improved prognoses, respond better to chemotherapy, and show decreased mortality and recidivism rates [138]. One research of 256 triple negative (TN) tumors found that the probability of recurrence decreased by 17% for every 10% increase in the number of TILs and a 27% reduction in the risk of mortality. Similarly, in 112 HER2 + BC patients, there was an 18% increase in overall survival (OS) for every 10% increase in the number of stromal TILs [139].

NFAT, NF-κB, and AP-1, three transcription factors with significant functions in T cells, are inhibited by B7-H3-Ig [140, 141]. In a mouse model, deletion of B7-H3 led to a significant reduction in the levels of other cosuppressor molecules, such as PD-1, and an increase in the production of the CD8 + T-cell proliferation markers Ki-67, IFN-γ, TNF-α, and granzyme B, indicating that B7-H3 is involved in the depletion of CD8 + T-cells [15]. NanoString data for tumor samples from triple-negative BC showed that in samples from the group with low TIL, B7-H3 was overexpressed [142]. One study evaluated the association between the percentage of TILs present and the expression of 800 genes associated with BC. The findings revealed that B7-H3 expression and the number of TILs were negatively associated [143]. In addition, our study also revealed that B7-H3 was negatively correlated with TIL levels in TNBC [144].

### Tregs

The regulation of inflammation is greatly influenced by T-regulatory (Treg) cells, which are crucial for immunological tolerance and homeostasis [145]. Given typical physiological circumstances, Treg cells are crucial in controlling the proliferation and activation of T and B cells and maintaining innate cytotoxic lymphocyte homeostasis [146]. Current research reveals that Treg-mediated immunosuppression is one of the primary ways that cancers subvert the immune system and a significant barrier to the effectiveness of tumor immunotherapy [147]. Treg cells suppress antitumor immunity via diverse mechanisms, including suppression of immune cells through direct contact and the production of regulatory cytokines [148]. Consistent with these observations, Treg cell depletion also evokes effective cancer immunity in tumor-bearing animals [149, 150]. The presence of Treg is associated with a more invasive BC phenotype and affects BC metastasis and prognosis [151].

The degree of B7-H3 expression and the presence of Tregs are positively correlated. In a B7-H3 deficiency model, the absolute number and proportion of Treg cells decreased [15]. Tumor cells may boost B7-H3 expression and encourage T cells to differentiate into Tregs. TNF-α and TGF-β1 production is thus elevated, which could promote immune evasion and the growth of tumor cells [152]. Moreover, the effect of Tregs in suppressing immune responses appears to be highly dependent on the expression of the transcription factor FOXP3, which regulates the expression of several genes that generate proteins that mediate Treg suppression, including CD25, GITR and CTLA-4 [153, 154]. FOXP3 is crucial for Treg function [155–157]. Treg cells that express FOXP3 are thus effective peripheral immunological tolerance mediators. B7-H3 expression and the quantity of FOXP3 + Treg cells have a strong positive connection [158], indicating that the recruitment of Treg cells may be a partial mediator of the immunosuppressive action of B7-H3.

### CAFs

Many stromal variables either repress or encourage genetic epithelial alterations to impact the complex ecosystems that makeup tumors. While normal fibroblasts suppress tumor formation [159], Cancer-associated fibroblasts (CAFs) promote tumor characteristics such as ECM remodeling, inflammation, and cancer cell proliferation and invasiveness [160–162]. It has been reported that different CAF populations produce various cytokine patterns in malignancies [163, 164]. CAFs produce alpha-smooth muscle actin (α-SMA) [165]. The development of several malignant tumors is strongly correlated with α-SMA expression [166, 167]. Increased stromal myofibroblasts in human BC are linked to aggressive adenocarcinomas and foretell disease recurrence [168]. Some tumor subtypes have also been linked to CAF subtypes, and CAFs that are positive for these CAF-associated markers have been predominantly found in HER2 and TNBC [169]. As mentioned earlier, BC often metastasizes to bone. It has been shown that CAFs play a crucial role in developing characteristics that enable cells in the original TME to metastasize to bone [170]. One study showed that primary tumor stroma enriched in CAFs could imitate the CXCL12-rich bone metastatic niche and promote the preselection of cancer cells that possess the potential to metastasize to bone [171].

Using an orthotopic xenograft tumor model they established in nude mice, Zhang et al. confirmed that B7-H3 + CAFs play a significant role in tumor growth and metastatic progression [172]. Another research revealed that the lack of B7-H3 reduced the release of cytokines, including interleukin (IL)-6 and vascular endothelial growth factor (VEGF), as well as the capacity of CAFs to migrate and invade [173]. In a subgroup of breast cancers, high B7-H3 expression on CAFs was shown to alter T-cell activity toward more regulatory activities [174]. Hence, more research is required into the role of B7-H3 expression in immune cell-connected fibroblasts.

The above observations, considered together, reiterate how crucial the immunological environment is for influencing clinical outcomes. Developing more effective treatment plans for BC will undoubtedly need combination therapy that targets both tumor cells and TME.

## B7-H3 as an attractive immunotherapy target

The ability to target B7-H3 via diverse effector pathways has recently been made available by developments in molecular biology and antibody design. Most of these tactics have been examined in mice and in vitro, and the testing has yielded safety and/or antitumor data, laying the foundation for clinical trials targeting B7-H3. It is regrettable that, as of now, no targeted drug has received FDA approval. Table 1 lists the current therapeutic studies being conducted to treat B7-H3.

**Table 1:** A list of the medications chosen for clinical trials against B7-H3 [175]

Trial ID	Drugs	Cancer types	Trial stage	Start date	Completion date	Recruitment status
**Targeting B7-H3 with monoclonal antibodies**						
NCT01391143	MGA271	Refractory cancer, melanoma, prostate, solid tumors	Phase l	July 2011	April 18, 2019	Completed
NCT02982941	MGA271	Pediatric patients with relapsed or refractory solid tumors	Phase l	December 2016	May 22, 2019	Completed
NCT02923180	MGA271	Prostate Cancer	Phase II	February 14, 2017	August 11, 2020	Active, not recruiting
NCT02381314	MGA271	Melanoma Non Small Cell Lung Cancer	Phase l	March 26, 2015	September 26, 2018	Completed
**Targeting B7-H3 with bispecific antibodies**						
NCT03406949	MGD009/MGA012	Relapsed/Refractory Cancer	Phase I	February 27, 2018	April 27, 2022	Completed
NCT02628535	MGD009	Mesothelioma and 11 other cancers	Phase I	September 2015	November 25, 2019	Terminated
**Targeting B7-H3 through ADC therapies**						
NCT03729596	MGC018	advanced solid tumors	Phase I/II	November 21, 2018	May 2023	Active, not recruiting
NCT02475213	MGA271 with pembrolizumab	Melanoma Head and Neck Cancer Non Small Cell Lung Cancer Urethelial Carcinoma	Phase I	July 2015	August 18, 2021	Completed
NCT04145622	DS-7300a	Advanced Solid Tumor, Malignant Solid Tumor	Phase I/II	November 3, 2019	December 1, 2023	Recruiting
NCT05280470	DS-7300a	Extensive-stage Small-cell Lung Cancer	Phase II	June 17, 2022	November 14, 2024	Recruiting
**Targeting B7-H3 with CAR T cells**						
NCT04185038	SCRI-CARB7H3	Ependymoma Germ Cell Tumor Diffuse Midline Glioma	Phase I	December 11, 2019	May 2041	Recruiting
NCT04077866	B7-H3 CAR-T	Recurrent Glioblastoma, Refractory Glioblastoma	Phase I/II	June 1, 2023	August 1, 2025	Recruiting
NCT04385173	B7-H3 CAR-T	Recurrent Glioblastoma, Refractory Glioblastoma	Phase I	December 1, 2022	May 1, 2024	Recruiting
NCT04483778	4-1BBζ B7H3-EGFRt-DHFR	Pediatric Solid Tumor, Germ Cell Tumor, Retinoblastoma	Phase I	July 13, 2020	December 2040	Recruiting
NCT04432649	4SCAR-276	Solid Tumor	Phase I/II	June 1, 2020	May 31, 2024	Recruiting
NCT05143151	CD276 CAR-T cells	Advanced Pancreatic Carcinoma	Phase I/II	July 1, 2021	July 2024	Recruiting
NCT05190185	TAA06	Malignant Melanoma, Lung Cancer, or Colorectal Cancer	Phase I	June 1, 2021	December 1, 2023	Recruiting
NCT04692948	TAA06	CAR Acute Myeloid Leukemia	Not Applicable	December 9, 2019	December 2023	Recruiting
NCT04637503	Combined 4SCAR-276	Neuroblastoma	Phase I/II	November 18, 2020	December 31, 2023	Recruiting
NCT04432649	4SCAR-276	Solid Tumor	Phase I/II	June 1, 2020	May 31, 2024	Recruiting
**Targeting B7-H3 with CAR NK cells**						
NCT03056339	AP1903	B-Lymphoid Malignancies	Phase I/II	June 21, 2017	June 30, 2024	Active, not recruiting
NCT04630769	MGA271/ FT516 and IL2	Ovarian cancer	Phase I	April 2, 2021	January 1, 2022	Recruiting
**Radioimmunotherapy**						
NCT01502917	124I-omburtamab	Brain cancer Brain Stem Glioma	Phase I	December 2011	January 2022	Completed
NCT01099644	131I-omburtamab	Peritoneal Cancer	Phase I	April 2010	September 2022	Active, not recruiting
NCT00089245	131I-omburtamab	Brain and Central Nervous System Tumors Neuroblastoma Sarcoma	Phase I	July 2004	July 1, 2025	Active, not recruiting
NCT03275402	131I-omburtamab	Neuroblastoma CNS Metastases Leptomeningeal Metastases	Phase II/III	December 11, 2018	December 2026	Recruiting
NCT05063357	131I-Omburtamab	DIPG	Phase I	March 2023	January 31, 2027	Not yet recruiting
NCT04022213	131I-Omburtamab	Desmoplastic Small Round Cell Tumor Peritoneal Cancer Peritoneal Carcinoma	Phase II	July 15, 2019	July 2024	Recruiting
NCT04743661	131I-omburtamab	Recurrent Medulloblastoma Recurrent Ependymoma	Phase II	April 4, 2022	October 30, 2029	Active, not recruiting
NCT04167618	177Lu-DTPA-omburtamab	Medulloblastoma, Childhood	Phase I/II	September 30, 2021	August 11, 2022	Terminated

### Targeting B7-H3 with monoclonal antibodies

Strong justification exists for using B7-H3-specific inhibitory monoclonal antibodies (mAbs) in the management of solid tumors due to the substantial alterations in cancer cells brought about by silencing of B7-H3 and the remarkable therapeutic outcomes of mAbs that block checkpoint molecules. It has been shown that using mAbs to block B7-H3 activity increases CD8 + T and NK cell tumor infiltration, prevents tumor growth, and/or lengthens life [176]. A mouse IgG1 mAb targeting B7-H3, 8H9, was shown to effectively against primary brain cancers [177]. 8H9 is currently being tested in phase 1 clinical studies to treat advanced CNS malignancies and desmoplastic small round cell tumors [178]. When the Fc part of an antibody interacts with immune cells to assault targets, the process is known as antibody-dependent cellular cytotoxicity (ADCC) [179]. Enoblituzumab (MGA271), a monoclonal antibody targeting the Fc region of B7-H3 with the potential to activate killer T cells through FcR binding, has demonstrated potent Antibody-Dependent Cellular Cytotoxicity (ADCC) against various xenograft tumors. It is currently undergoing clinical trials for the treatment of resistant malignancies (NCT02982941, NCT02923180, NCT02381314, NCT04630769, NCT02475213 and NCT01391143) [180].

### Targeting B7-H3 with bispecific antibodies

Nisonoff and his colleagues originally introduced the idea of a bispecific antibody (bsAb), a synthetic antibody-based molecule with two distinct antigen-binding sites, more than 60 years ago [181]. The ensuing conceptual and technical developments in the production of bsAbs evolved in tandem with groundbreaking developments in antibody design and physiology disciplines [182]. BsAbs’ ability to allow dual-targeting ideas holds significant therapeutic potential. For example, the anti-CD3 mAb scFv was combined with the anti-B7-H3 mAb scFv to create obrindatamab [183]. Obrindatamab instructs T lymphocytes to attack B7-H3 + tumor cells by attaching simultaneously to CD3 and B7-H3. Obrindatamab demonstrated an enhancement in T-cell cytotoxicity by stimulating the production of IL-2, TNF-α, and IFN-γ. This resulted in a substantial reduction in tumor development, leading to increased survival in immunodeficient animals [183]. The B7-H3-targeting bispecific antibody now undergoing clinical review, is being investigated for its potential synergy with anti-PD-1 treatment, although no results have been made public as of yet. Recently, Huang et al. created a BiTE-based mRNA therapy by encasing the mRNA that codes for B7-H3CD3 BiTE inside brand-new ionizable lipid nanoparticles (LNPs). These findings imply that treatment approaches based on B7-H3 × CD3 BiTE mRNA expression may be beneficial and have good clinical application possibilities [184].

### Targeting B7-H3 through ADC therapies

Antibody–drug conjugates (ADCs), hybrid molecules designed for targeted therapy, have demonstrated considerable promise in facilitating a paradigm change in cancer therapy through antibody-antigen interactions [185]. ADCs comprise a potent cytotoxic payload, a humanized antibody that targets tumors, and a linker that connects them [186]. Antibody–drug conjugation systems are sophisticated, cutting-edge strategies that can deliver the best outcomes in BC therapy. MGC018 is a DNA-alkylating anti-B7-H3 ADC that has been studied in phase 1 dose-expansion trials and has been shown to have robust anticancer efficacy in various cancer models (NCT03729596) [187]. In a more recent clinical study, DS-7300a, an ADC that combines a humanized anti-B7-H3 antibody that contains an inhibitor of DNA topoisomerase I, has shown to be secure and reliable in the treatment [188]; the published interim results show good tolerability in patients with advanced tumors. Scientists have been immensely enthused by the DS-7300a’s early achievements, and a fresh trial testing DS-7300a’s efficiency has started (NCT05280470).

### Targeting B7-H3 with CAR T cells and CAR NK cells

Two types of immune cells, CD8 + cytotoxic T and NK cells, destroy their target cells through similar cytotoxic processes. While HLA class I antigen expression is not required to detect tumor cells by Chimeric Antigen Receptor (CAR) T cells, the CAR T cells detect tumor cells quickly and with solid cytotoxicity [189]. B7-H3 CAR T cells with different B7-H3-specific scFvs exhibit potent in vitro antitumor efficacy against several tumor types [190–193]. In the case of reports, B7-H3-targeted CAR-T cells exhibited excellent tolerance in patients with relapsed basal cell carcinoma, glioblastoma, and recurrent anaplastic meningioma [194]. Combinatorial approaches that increase CAR-T cell antitumor efficacy and the vulnerability of tumor cells to the effector mechanism are being studied. Regarding cost-effectiveness, while CAR-T therapy has shown remarkable clinical outcomes, its economic implications, including manufacturing costs, accessibility, and long-term sustainability, need careful consideration.

As a crucial component of the innate immune response against malignancy, NK cells are capable of directly destroying tumors [195]. Nonetheless, it has been demonstrated that the cytotoxicity of NK cells is functionally compromised by the immunosuppressive characteristics of B7-H3 in several cancers [196]. It is possible to obtain CAR with distinct specificity for cancer immunotherapy and use it to enhance NK cell function in malignancy. Several clinical scenarios have demonstrated the superior safety of CAR-NK cell immunotherapy and shown that it has a lower risk of causing neurotoxicity and cytokine release syndrome [197, 198]. Findings from the first large-scale study using CAR-NK cells in individuals with CD19 + chronic lymphocytic leukemia and B-cell lymphoma demonstrated safety and showed encouraging clinical efficacy [199]. Tumor heterogeneity, the disappearance of the targeted antigen, and antagonistic TME are the insurmountable difficulties that CAR-NK cell therapy now confronts. Several strategies should be taken into consideration in the future to optimize the efficacy of CAR-based NK cell treatment.

### Radiotherapy

Radioimmunotherapy slows tumor growth by attaching radionucleotides to tumor-targeting antibodies, producing radiation-induced cytotoxicity [199]. The carrier most often utilized in radioimmunoconjugates is omburtamab. In phase I trials, intrathecal omburtamab was well tolerated by patients treated for metastatic central nervous system neuroblastoma and intraperitoneal 131I-mAb 8H9 in desmoplastic small round cell tumors (NCT04022213) [200]. Delivering 124I-mAb 8H9 to diffuse pontine glioma through convection-enhanced brainstem caused low systemic exposure and no harm (NCT01502917) [175]. Control of radiotoxicity remains a significant obstacle that must be overcome when attempting to treat other solid tumors using radioimmunotherapy against B7-H3.

### B7-H3 small-molecule inhibitors

By combining computational modeling with an in silico technique, synthetic chemical libraries can be screened to identify compounds with apparent inhibitory effects on B7-H3. These compounds provide various observable advantages; their small size and solubility allow them to readily cross membrane barriers such as the blood–brain barrier, allowing precise penetration into different tissues, including TMEs. This makes them particularly helpful for the treatment of central nervous system cancers. Compared to antibody-based or CAR therapy, the cost of producing small-molecule inhibitors is minimal, and the conditions required for their storage are less rigorous [201]. Thus, targeting B7-H3 with small-molecule inhibitors might be an appealing alternative or supplementary treatment approach.

## Application of B7-H3 in tumor imaging

B7-H3 has shown promise for therapeutic use in tumor imaging in addition to being a prognostic marker and an immunotherapy target. The first line of defense in BC screening programs is mammography. The median size of lesions identified with mammography screening is 1.5 cm; however, the median size identified through clinical detection is 2.6 cm [202], and digital mammogram analysis greatly boosts screening sensitivity [203]. Unfortunately, mammograms frequently lead to overdiagnosis and pointless biopsies, and half of the women who receive multiple screenings report experiencing false-positive results [204].

It has been established that B7-H3 is a target for BC molecular ultrasound imaging. As molecular targeting contrast agents, microbubbles functionalized with B7-H3-targeted affibodies [205] or antibodies [206] have shown excellent promise. While nontargeted microbubbles produced lower imaging signals in normal mammary tissues and malignancies that block B7-H3, Strong imaging signals were obtained in tumors expressing hB7-H3 by microbubbles conjugated to the B7-H3-targeted affibody (MBABY-B7-H3) [205], proving the B7-H3’s diagnostic utility in BC imaging. Spectroscopic photoacoustic imaging is a new focused approach [207]. Using an affibody or antibody that is specific for B7-H3 and conjugated to indocyanine green, researchers can detect BC [208], assess the tumor’s grade [209], and direct the resection during surgery.

## Conclusion

BC is the primary cancer-related killer of women worldwide and is regarded as a lethal malignant tumor in most countries. The threat of BC lies not only in its widespread incidence but also in its cunning ability to relapse and metastasize. The BC patient’s treatment journey is often accompanied by multiple treatment modes such as surgery, radiotherapy, chemotherapy. Given the strain on the patient’s body and the fact that conventional procedures may not always appear sufficient, new effective and gentle therapeutic approaches are especially required.

Within this context, the stable high expression of B7-H3 in a variety of cancers is of great interest to researchers, especially in BC. The close correlation between elevated expression levels of B7-H3 and an unfavorable prognosis provides compelling evidence for its potential as a promising therapeutic target. Furthermore, preclinical studies and early trials have also shown the value of B7-H3 as a serum marker for use in BC diagnosis and prognosis. Its integration into breast ultrasound imaging further underscores its potential as a non-invasive tool for early disease detection and monitoring.

Overall, while B7-H3 shows promise in BC treatment and may serve as a therapeutic target, continued research is needed to fully understand its complex receptor interactions and overcome barriers to developing potent B7-H3 inhibitors. By overcoming these challenges, new therapeutic approaches may be developed, instilling renewed hope in BC patients worldwide.

## Data Availability

No datasets were generated or analysed during the current study.

## Abbreviations

BC: Breast cancer
HR +: Hormone receptor-positive
ER +: Estrogen receptor alpha-positive
PR +: Progesterone receptor
HER2 +: Human epidermal growth factor receptor 2 positive
TNBC: Triple negative breast cancer
ICI: Immune checkpoint inhibitor
PD-1: Programmed death 1
TME: Tumor microenvironment
APCs: Antigen-presenting cells
IgV: Variable
IgC: Constant
sB7-H3: Soluble B7-H3
BBD: Benign breast disease
CSCs: Cancer stem cells
BCSCs: Breast cancer stem cells
EMT: Epithelial-mesenchymal transition
ROS: Reactive oxygen species
MDSCs: Myeloid-derived suppressor cells
NK: Natural killer
TAMs: Tumor-associated macrophages
ECM: Extracellular matrix
Th1: T-helper 1
FOXP3: Forkhead Box P3
Th2: T-helper cells
CTL: Cytotoxic T-cells
TILs: Tumor-infiltrating lymphocytes
TN: Triple negative
OS: Overall survival
Treg: T-regulatory
CAFs: Cancer-associated fibroblasts
α-SMA: Alpha-smooth muscle actin
VEGF: Vascular endothelial growth factor
mAbs: Monoclonal Antibodies
ADCC: Antibody-dependent cellular cytotoxicity
bsAb: Bispecific antibody
LNPs: Lipid nanoparticles
ADCs: Antibody–drug conjugates
CAR: Chimeric Antigen Receptor
